# Hierarchical Information-guided robotic grasp detection

**DOI:** 10.1038/s41598-025-03313-z

**Published:** 2025-05-29

**Authors:** Zeyao Hou, Yueran Zhao, Yutao Jin, Chao Yang, Zongyu He, Xiaoyan Chen

**Affiliations:** 1 Tianjin University of Science and Technology, Tianjin, 300222 China; 2 Tianjin College, University of Science and Technology Beijing, Tianjin, 301830 China; 3 Tianjin Bonus Robotics Technology Co., Ltd., Tianjin, China

**Keywords:** Deep Learning, Robotic Grasp Detection, Transformer, Attention Mechanism, Mathematics and computing, Computer science

## Abstract

With the advancement of deep learning, robotic grasping has seen widespread application in fields, becoming a critical component in enhancing automation. Accurate and efficient grasping capabilities not only significantly boost productivity but also ensure safety and reliability in complex and dynamic environments. However, current approaches, particularly those based on convolutional neural networks (CNNs), often neglect the hierarchical information inherent in the data and lead to challenges in complex environments with abundant background information. Moreover, these methods struggle to capture long-range dependencies and non-local self-similarity, critical for accurate grasp detection. To address these issues, we propose GraspFormer, a novel method for robotic grasp detection. GraspFormer features a unique Encoder-Decoder framework that incorporates a Grasp Transformer Block designed to model long-range dependencies while avoiding background interference. Our approach also designs hierarchical information-guided self-attention (HIGSA) and an adaptive deep channel modulator (DCM) to enhance feature interactions and competition. Extensive experiments demonstrate that GraspFormer achieves performance comparable to state-of-the-art methods. The code is available at https://github.com/shine793/Hierarchical-Information-guided-Robotic-Grasp-Detection.

## Introduction

In recent years, the rapid development of artificial intelligence has significantly elevated the importance of intelligent robots across various industries. These robots have been increasingly integrated into fields such as manufacturing^1^, healthcare^2^, agriculture^3^and logistics^4^, where they play a pivotal role in enhancing efficiency and precision. Among the many capabilities of these robots, the ability to grasp objects stands out as a crucial factor in their ability to perform complex tasks. Effective robotic grasp detection, therefore, is fundamental to enabling robots to interact with and manipulate their environment in a manner that is both safe and reliable.

Current approaches to robotic grasp detection can be broadly categorized into two main types: analytical methods and data-driven methods. Analytical methods rely on mathematical models and physical principles to predict stable grasps. These methods often provide robust performance in well-defined environments and offer interpretable results due to their basis in established theories. However, they can struggle with generalization to complex, unstructured settings, where the assumptions of the models may not hold. On the other hand, data-driven methods, typically leveraging deep learning, have shown remarkable adaptability and performance in diverse scenarios by learning from large datasets. Although data-driven methods may lack interpretability and demand significant computational resources, their advantages in adaptability and performance have led to a growing number of research efforts favoring these approaches.

To achieve robotic grasp detection, researchers have developed numerous convolutional neural networks (CNNs) for vision-based robots. However, these approaches typically employ CNNs to directly learn a brute-force mapping function from input samples to grasp outcomes, often overlooking the hierarchical information inherent in the data. This oversight can lead to challenges in complex environments, where background information is often more abundant than the target object, potentially interfering with the grasp detection process. Furthermore, these CNN-based methods show limitations in capturing long-range dependencies and non-local self-similarity, which are critical for robotic grasp detection. While introducing recently rising deep learning model, Vision Transformer (ViT), may provide a possibility to address the problems of CNNs-based approaches. Nevertheless, directly utilizing original ViT for robotic grasp detection may encounter the drawback that its computational complexity is quadratic to the input spatial size. Thus, the potential of Transformer for robotic grasp detection still remains under-explored.

To deal with these problems, we propose a novel method, GraspFormer, for robotic grasp detection. Specifically, we formulate a Encoder-Decoder framework which combines a novel Transformer designs named Grasp Transformer block (GTB). It utilizes the estimated shallow information and current information to avoid the influence of background features and model the long-range dependencies. The key component of GTB is hierarchical information-guided self-attention (HIGSA). HIGSA exploits the hierarchical representations to direct the computation of self-attention and enhance the interactions between different regions. Different from previous Transformer-based robotic grasp detection solutions, we further explore the importance of channel information in the high-dimensional features. We devise an adaptive deep channel modulator (DCM) to encourage feature competition between channels. Extensive experiments and ablation studies are conducted to demonstrate that the effectiveness of the proposed GraspFormer and it achieves comparable results among several state-of-the-art methods.

Our contributions are summarized as follows:

- We propose a novel framework dubbed GraspFormer for robotic grasp detection.
- We design a new hierarchical information-guided self-attention mechanism, which utilizes the hierarchical features to guide the modeling of long-range dependences.
- We formulate an adaptive deep channel modulator to encourage channel competition, improving the feature representation in high-dimensional space.
- Extensive experiments are conducted to show that GraspFormer achieves better performance among different state-of-the-art robotic grasp detection solutions on representative public datasets.

## Related work

Grasp detection is a widely studied task in both the computer vision and robotics communities^5–7^.Most works are based on vision-based grasp detection approaches and analytical methods.

**Vision-based grasp detection approaches.** Vision-based grasp detection for robot manipulation involves utilizing visual data, usually captured by cameras or imaging devices, to determine optimal grasping positions on objects^8–11^. Guo et al.^12^propose a convolutional neural network to generate grasp candidates and produce predictions by evaluating the feature vectors of the candidate regions. Zhou et al.^13^ proposed a framework that predicts multiple grasping poses through the use of oriented anchor boxes. Morrison et al.^14^proposed the Generative Grasp CNN (GG-CNN) to perform the grasp task, which inspired the development of several other grasp detection models. However, these methods heavily relies on depth information and is vulnerable to environmental interference, necessitating higher standards for image collection. Furthermore, these approaches are primarily based on various convolutional neural networks^15–18^, which have limitations in capturing non-local information. In recent years, transformers have gained significant attention in computer vision due to their ability to capture global context, addressing the limitations of traditional CNN models. They have demonstrated outstanding performance in tasks such as object detection, classification, and tracking^14,19^, largely thanks to their self-attention mechanism and hierarchical structure. Zhang et al.^20^ utilized the Swin Transformer to achieve remarkable feature extraction results. However, while the self-attention mechanism is effective, it primarily focuses on information within individual samples, overlooking the hierarchical information extracted from images. This could potentially reduce the robustness of feature representations.

**Analytical Method.** Analytical methods often rely on monocular depth estimation, which is a key task in computer vision that involves predicting depth information from a single 2D image captured by a monocular camera. Accurate depth data is essential for understanding the three-dimensional structure of a scene. The field of monocular depth estimation began with the pioneering work of Saxena et al.^21,22^, which employed hand-crafted features and Markov Random Fields (MRF). In the subsequent development, Kendall et al.^23^ studied the role of uncertainty estimation in scene understanding, and Yin et al.^24^ utilized surface geometry to estimate 3D point clouds from depth maps. However, these methods often lose the fine details, and their performance typically lacks robustness.

## Method

**Fig. 1:**
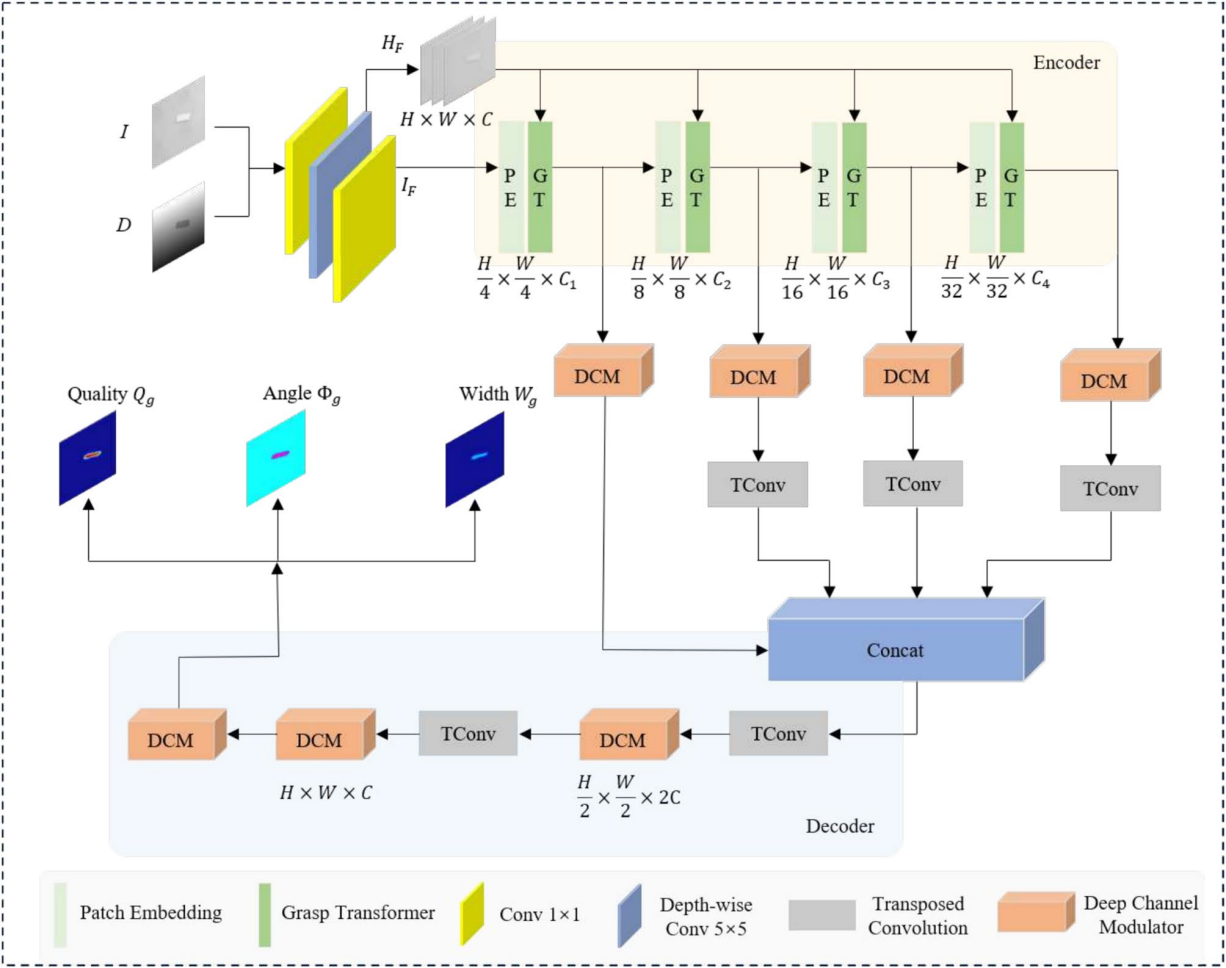
The overview of our method, which is a encode-decoder framework.

### Overview

In vision-based grasping tasks, the process usually begins with capturing visual images of the target object using sensors such as RGB-D cameras. These images undergo processing through a model that determines the most effective grasp position. When the robot utilizes parallel grippers, the grasping parameters *p* are typically expressed as a 5-dimensional tuple, encompassing various critical aspects necessary for executing a successful grasp, which can be expressed as,

1 $$\begin{aligned} p=\left\{ x,y,\theta ,w,h \right\} \end{aligned}$$

where (*x*, *y*) denotes the 2-dimensional coordinates of the center point, (*w*, *h*) is the width and height of the grasping box, $\theta$ represents the rotation angle of the gripper relative to the horizontal axis.

To achieve high-precision, real-time robot grasping, based on^14^, the grasp task is redefined for 2DoF robotic grasping problems as,

2 $$\begin{aligned} p=\left\{ Q_{g} ,\Phi _{g},W_{g} \right\} \in \mathbb {R} ^{3\times W\times H} \end{aligned}$$

where $Q_g$ indicates grasp success of each pixel, $\Phi _{g}$ corresponds to the orientation angle between the gripper’s fingertips, $W_g$ specifies the opening width between the fingertips of the gripper. For each pixel, the values $\Phi _{gi,j}$ and $W_{gi,j}$ reflect the specific orientation angle and width of the gripper’s fingers at that location. Additionally, *W* and *H* represent the height and width of the feature map, respectively.

The overall architecture of the proposed GraspFormer is presented in Figure 1. GraspFormer consists of the encoder, decoder and prediction head. The basic unit of GraspFormer is GraspTransformer block (GTB). Given an input image *I* and its depth image *D*, we first use the hierarchical information estimator $\varepsilon$ with convolutional operators to outputs a hierarchical feature $H_{f}\in \mathbb {R} ^{H\times W\times C}$ and image feature $I_{f}\in \mathbb {R} ^{H\times W\times C}$ as,

3 $$\begin{aligned} \left( H_{f},I_{f} \right) =\varepsilon \left( I,D \right) \end{aligned}$$

where $I_{f}$ is fed to the subsequent framework and $H_{f}$ is processed to the other transformers. For the encoder, to transform the given samples into sequence embeddings, the given inputs are split into non-overlapping patches by the patch embedding. Then, several Grasp Transformer block produce multi-level features with resolution of $\left\{ 1/4, 1/8, 1/16, 1/32 \right\}$ of the original sample. These features are then up-sampling by the transparent convolutional operator. By introducing the designed adaptive deep channel modulator (DCM), the four levels of features are calibrated. To ensure that the height and width of the obtained features match the original data, two transposed convolution are employed to up-sample the features. This process adjusts the dimensions of the features so that they align accurately with the input data. For the decoder, we utilize three DCMs to enhance the robustness of the features in the last parts. After that, we use the prediction head to predict quality, angle and width heatmaps.

**Fig. 2:**
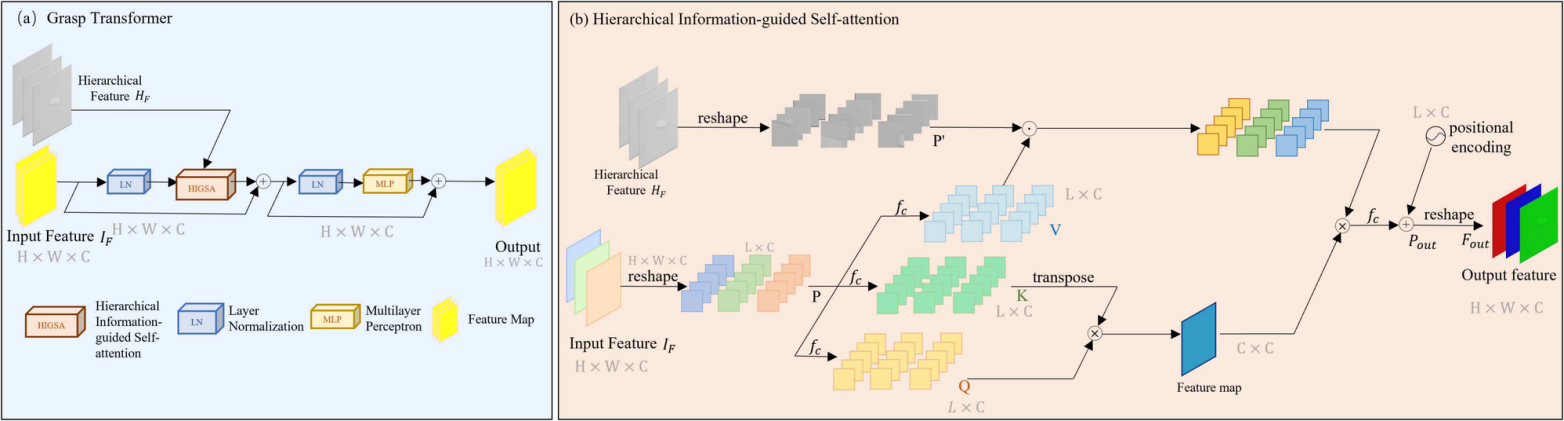
The illustration of the proposed Grasp Transformer and Hierarchical Information-guided Self-attention.

### Grasp transformer

Previous work in robotic grasp detection primarily relies on CNNs, which have limitations in capturing non-local dependencies. While Transformers could address these limitations, directly applying them introduces substantial computational complexity and ignore the hierarchical information. The full potential of Transformers remains underexplored. To address this, we propose a Grasp Transformer to perceive the long-range features.

**Structure.** As shown in Figure 2(a), Grasp Transformer includes two residual sub-block. The first sub-block contains a layer normalization and a hierarchical information-guided self-attention (HIGSA). Note that HIGSA needs the hierarchical feature $H_{f}$ to perceive information. The second sub-block includes a layer normalization and a multilayer perceptron. Each of them also contains a residual connection to avoid losing information. Given the input feature *F*, the output $F^{ ' }$ of Grasp Transformer can be expressed as,

4 $$\begin{aligned} S&=F+ HIGSA\left( LN\left( F \right) ,H_{f} \right) \end{aligned}$$

5 $$\begin{aligned} F '&=S + MLP(LN(S)) \end{aligned}$$

where $LN(\cdot )$ denotes a layer normalization, and $MLP(\cdot )$ refers a multi-layer perceptron.

**Hierarchical Information-guided Self-attention.** As shown in Figure 2(b), the hierarchical feature $H_{f}$ estimated by the estimator $\varepsilon$ is transferred into each HIGSA of Grasp Transformer. Please see that Figure 2(b) illustrates HIGSA for the largest scale. For smaller scales, we use convolution with size of $4\times 4$ and stride=2 to downscale *H* to match the spatial size, which is omitted in that figure.

Firstly, the given input feature $F\in \mathbb {R} ^{H\times W\times C}$ is reshaped into patch tokens $P\in \mathbb {R} ^{L\times C}$, where $L=H\times W$ denotes the sequence length. Then *P* is split into *k* heads:

6 $$\begin{aligned} P=\left\{ P_{1},P_{2},\dots ,P_{k} \right\} \end{aligned}$$

where $P_{i}\in \mathbb {R} ^{L\times d_{k} }$, $d_{k} =C/k$, and $i=1,2,\dots ,k$. Figure 2(b) is the situation with $k=1$. For each head, we use three connected layers $f_{c}$ without bias to project and transform $P_{i}$ into query elements $Q_{i}\in \mathbb {R}^{L\times d_{k} }$, key elements $K_{i}\in \mathbb {R}^{L\times d_{k} }$, and value elements $V_{i}\in \mathbb {R}^{L\times d_{k} }$) as

7 $$\begin{aligned} Q_{i}=P_{i}W_{Q_{i} }^\top ,K_{i}=P_{i}W _{K_{i} } ^\top ,V_{i}=P_{i}W_{V_{i} }^\top \end{aligned}$$

where $W_{Q_{i} }$, $W_{K_{i} }$, and $W_{V_{i} } \in \mathbb {R} ^{d_{k}\times d_{k} }$ denote the learnable parameters of the connected layers $f_{c}$ and $\top$ is the matric transpose. We observe that different regions of the same image may contain distinct contextual information, such as foreground objects and background scenes. The foreground, often where the grasping targets are located, requires precise detection and is more challenging to analyze due to its complexity. Background regions, with less complex information, can provide contextual cues to assist in accurately identifying and enhancing the detection of foreground objects. Thus, we use the hierarchical information $H_f$ encoding and interacting different regions to direct the computation of self-attention, To match the shape of *P*, we reshape $H_f$ into $P ' \in \mathbb {R}^{L\times C}$ and split it into *k* heads,

8 $$\begin{aligned} P '=\left\{ P_{1}^{\prime } ,P_{2}^{\prime },\dots ,P_{k}^{\prime } \right\} \end{aligned}$$

where $P_{i}^{\prime } \in \mathbb {R}^{L\times d_{k} }$,$i- 1,2,\dots ,k$.For each head, the self-attention can be formulated as,

9 $$\begin{aligned} Attention\left( Q_{i},K_{i},V_{i},P_{i}^{\prime } \right) =\left( P_{i}^{\prime }\cdot V_{i} \right) softmax\left( \frac{K_{i}^{T}Q_{i} }{\alpha _{i} } \right) \end{aligned}$$

**Table 1:** Comparison with transformer. The best results are boldfaced.

Approaches	Accuracy (100%)		Complexity	
	Image-wise	Object-wise	Params (M)	FLOPs (G)
Transformer	97.88	96.52	68.25	73.91
Ours	**98.91**	**97.30**	**1.57**	**15.14**

where $\alpha _{i}$ is a learnable parameter that adaptively scales the matrix multiplication, and $\cdot$ is the matrix multiplication. Next, *k* heads are concatenated and passed through a fully connected layer $f_{c}$, followed by the addition of a positional encoding which consists of learnable parameters to generate the output token $P_{out} \in \mathbb {R}^{L\times C}$. Finally, $P_{out}$ is reshaped to produce the output feature $F_{out} \in \mathbb {R}^{H\times W\times C}$.

**Complexity Analysis.** In this section, we analyze the computational complexity in our HIGSA arises from the *k* computations involved in the two matrix multiplications described in Equation 9, *i*.*e*., $\mathbb {R} ^{d_{k}\times L }\times \mathbb {R}^{L\times d_{k}}$ and $\mathbb {R}^{L\times d_{k}} \times \mathbb {R}^{d_{k}\times d_{k}}$. Thus, the complexity $\vartheta$(HIGSA) can be expressed as,

10 $$\begin{aligned} \begin{aligned} \vartheta \left( HIGSA \right)&=k\times \left[ d_{k}\times \left( d_{k}\times L \right) + L\times \left( d_{k}\times d_{k} \right) \right] \\&=2Lkd_{k}^{2} =2Lk\left( \frac{C}{k} \right) ^{2} =\frac{2LC^{2} }{k} \end{aligned} \end{aligned}$$

For previous global multi-head self-attention (MSA), its complexity is,

11 $$\begin{aligned} \vartheta \left( MSA \right) =2L^{2} C \end{aligned}$$

Through the above analysis, it can be observed that $\vartheta$(MSA) is quadratic to the sequence size *L*. It’s burden is expensive and limits their application of Transformer for robotic grasp detection. Compared with previous MSA, our method is linear to the sequence length, HIGSA has lower computational complexity enables our Grasp Transformer to be plugged into our framework. As shown in Table 1, we conduct a verification on Cornell dataset. We introduce a transformer block replace the Grasp Transformer, and find that our method reaches competitive performance with only about 2% parameters of the transformer.

### Deep channel modulator

To facilitate the generation of multi-scale features and increase the selectivity of channels, we design a deep channel modulator (DCM) to encourage the contrast of channels. For the input feature *F*, DCM processes it by global feature aggregation and feature calibration. Specifically, we first aggregate the *F* into a vector $g_f$ with a global function $G\left( \cdot \right)$ as $G\left( F \right) : F\in \mathbb {R} ^{H\times W\times C}\mapsto \mathbb {R}^{C}$. It can be achieved by a pooling layer. Next, we introduce *L*2-norm to attain a set of aggregated values $G\left( F \right) =g_f= \left\{ \left\| F_{1} \right\| , \left\| F_{2} \right\| ,\dots ,\left\| F_{c} \right\| \right\} ^{\textrm{T}} \in \mathbb {R} ^{C}$ is a scalar that aggregates the statistics of the i-th channel. Then, we employ a normalization function $NF\left( \cdot \right)$ to the aggregated values, which can be formulated as $NF\left( \left\| F_{i} \right\| \right) : \left\| F_{i} \right\| \in \mathbb {R}\mapsto \frac{\left\| F_{i} \right\| }{\sum _{j=1,2,\dots ,C} \left\| F_{j} \right\| }\in \mathbb {R}$.

To easy optimization, we apply two additional learnable parameters $\alpha$ and $\beta$, and initialize them to zero. To avoid feature loss, we also apply a skip connection between the input and output of the DCM. The resulting final DCM is $F_{i}= \alpha \times NF\left( G\left( F \right) _{i} \right) +F_{i}+ \beta$.

### Loss function

In this research, the robotic grasp detection task is implemented using a one-stage framework. Moreover, the smooth $L_{1}$ loss function is employed as the optimization target, offering robustness against outliers and ensuring stability throughout the training process. The loss can be written as,

12 $$\begin{aligned} L\left( T_{\gamma }, T_{\gamma }^{\prime } \right) =\sum _{\gamma \in \left\{ q,\theta ,w \right\} } Smooth_{L_{1} } \left( T_{\gamma }^{\prime }- T_{\gamma } \right) \end{aligned}$$

where $T_{\gamma }^{\prime }$ is the ground truth, $T_{\gamma }$ represents predicted grasping parameters, $q,\theta$, and *w* denotes the grasping quality, rotation angle, and the opening width between the fingertips of the gripper, respectively. The $Smooth_{L_{1} } \left( T_{\gamma }^{\prime }- T_{\gamma } \right)$ can be defined as,

13 $$\begin{aligned} Smooth_{L_{1}}\left( T_{\gamma }^{\prime }-T_{\gamma }\right) =\left\{ \begin{array}{l} 0.5\left( T_{\gamma }^{\prime }-T_{\gamma }\right) ^{2},\left| \left( T_{\gamma }^{\prime }-T_{\gamma }\right) \right| <1 \\ \left| \left( T_{\gamma }^{\prime }-T_{\gamma }\right) \right| -0.5,otherwise \end{array}\right. \end{aligned}$$

## Experiment

### Dataset

In this section, all experiments are conducted on Cornell^25^ and Jacquard^26^ datasets. The following parts show the datasets details, implementation and evaluation index.

**Datasets.** Cornell dataset, released in 2013, contains 240 unique objects with 885 color and 885 depth images. To optimize performance with the transformer architecture, which demands large datasets, we applied data augmentation techniques such as rotation, scaling, and random cropping. Jacquard dataset includes 54,485 diverse scenes featuring 11,619 distinct objects, offering RGB images, 3D point clouds, and grasp annotations. Due to the vast scale of the Jacquard dataset, no data augmentation methods were utilized in this study.

**Implementation.** We develop our framework using the PyTorch platform. The training process leveraged an NVIDIA GTX 1080 Ti GPU. For data augmentation on the Cornell dataset, each $640 \times 480$ image was rotated, scaled, and randomly cropped, producing a final image size of $224 \times 224$. The Jacquard dataset has a resolution of $640 \times 480$, which we resized to $224 \times 224$. During training, images were randomly sampled from the dataset, with each epoch comprising 200 batches of 32 images, and the model was trained for a total of 100 epochs. The AdamW optimizer was used to train our network, with an initial learning rate set to 0.0001. The initial learning is halved every 25 epochs. The model was configured with specific parameters: the channel counts were set to $2^{5}$, $2^{6}$, $2^{7}$, and $2^{8}$ respectively. The number of heads in the external attention layers was set to 1, 2, 4, and 8, respectively. For the number of channels in decoder, we set $C=2^{8}$

The datasets were divided into two parts, with 90% allocated for training and 10% for testing. The performance of our GraspFormer was evaluated using both image-wise and object-wise detection accuracy. In the image-wise split, the dataset was randomly divided 9:1 to assess the model’s ability to generalize previously seen objects in various contexts and orientations. In contrast, the object-wise split separated the dataset by object instances, ensuring that no identical objects appeared in both the training and testing sets, thus evaluating the model’s capability to generalize to unseen objects.

**Evaluation index.** The predicted grasping box is deemed accurate if it satisfies the following angle and Intersection over Union (IOU) criteria:

- The angle error between the predicted and labeled values must be within $\frac{\pi }{6}$.
- The IOU metric, as defined in the following equation, must exceed 0.25.

14 $$\begin{aligned} IOU\left( R^{*}, R\right) =\frac{\left| R^{*} \cap R\right| }{\left| R^{*} \cup R\right| } \end{aligned}$$

### Comparison studies

**Fig. 3:**
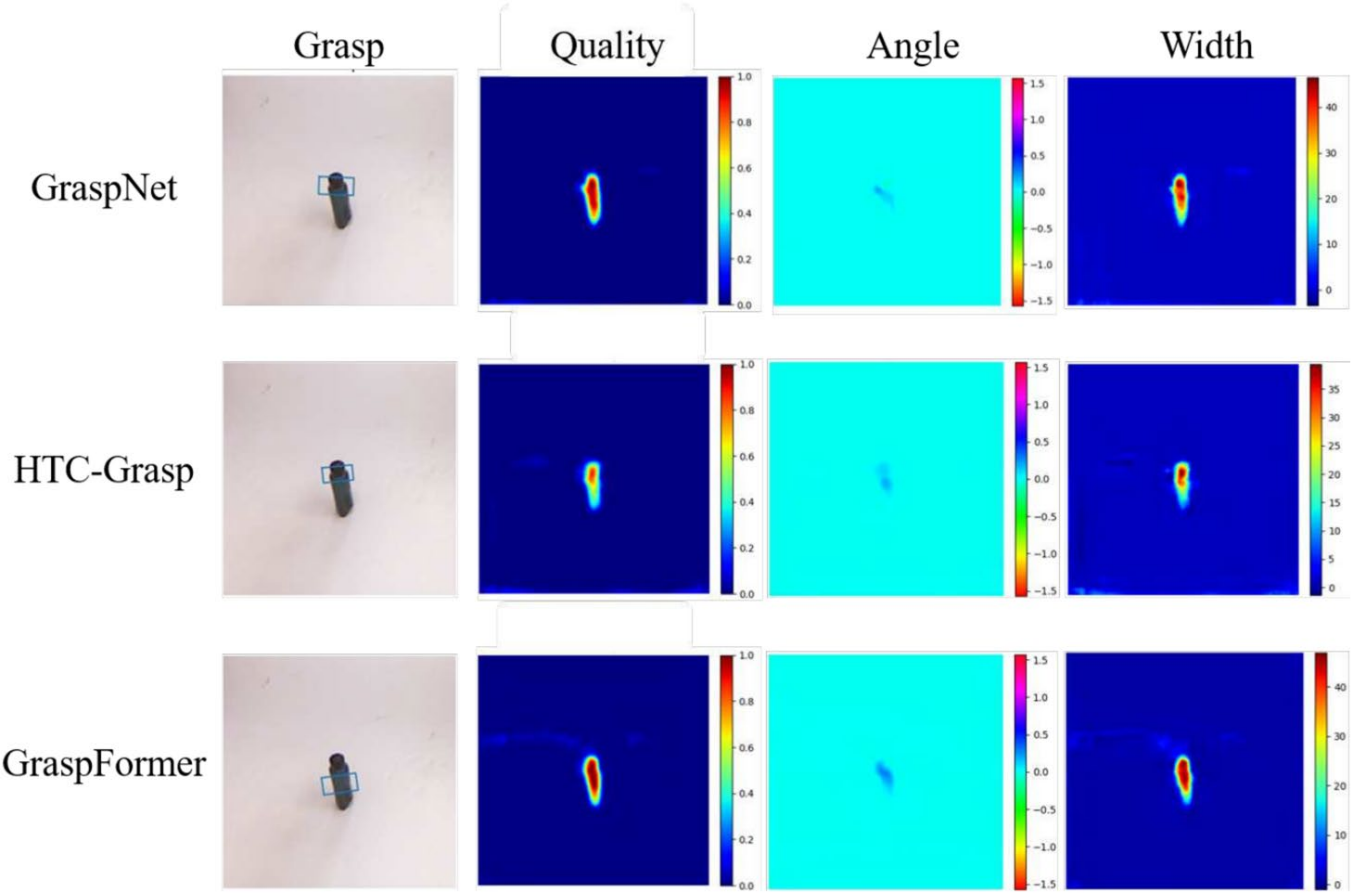
Comparison of the predicted results and heat maps on Cornell dataset. The columns represent RGB inputs, depth images, grasping rectangles, quality, angle and width heat maps. Note that the bounding boxes are included in the grasp map.

To compare our method with other state-of-the-art grasp detection methods, the same evaluation metrics were applied. The comparison begins with an analysis using the Cornell dataset. We compare our method with a rich collection of SOTA methods for robotic grasp detection on the Cornel dataset, including Fast Search^25^, GG-CNN^14^, SAE^27^, Two-stage closed-loop^28^, MultiGrasp^29^, STEM-CaRFs^30^, GRPN^31^, ResNet-50x2^32^, GraspNet^33^, ZF-net^12^, E2E-net^34^, HTC-Grasp^35^, GR-ConvNet^36^ and TF-Grasp^37^. We also conduct experiments on Jacquard dataset with several SOTA methods including Jacquard^26^, GG-CNN2^14^, FCGN^13^, GQ-STN^38^, ROI-GD^39^, Det Seg^34^, GR-ConvNet^36^, and HTC-Grasp^35^. Grasp positions are determined through four output heatmaps, where the center pixel at the most probable grasp location has the highest predicted quality value. The size and rotation of the grasping rectangle are obtained by indexing the other three parameters corresponding to this center pixel. Figure 3 shows the results of GraspNet^33^, HTC-Grasp^35^, and the proposed GraspFormer for unseen objects on the Cornell dataset. Statistical analysis in Table 2 demonstrates that GraspFormer delivers superior grasp quality compared to other methods. For the experimental results of classical methods presented in Table 2, the data reported in their original papers were used. This table highlights GraspFormer’s performance in comparison to existing algorithms on the Cornell dataset, showing that GraspFormer outperforms other methods with accuracy rates of 98.91% for image-wise tests and 97.30% for object-wise tests.Additionally, GraspFormer processes a single frame in about 5.1 ms using the NVIDIA GTX 1080 Ti GPU, meeting the requirements for real-time processing.

**Table 2:** The accuracy on Cornell dataset.

Method	Input	Accuracy(%)		Time(ms)
		IW (Image-Wise)	OW (Object-Wise)	
Fast Search^25^	RGB-D	60.5	58.3	5000
GG-CNN^14^	D	73.0	69.0	19
SAE^27^	RGB-D	73.9	75.6	1350
Two-stage closed-loop^28^	RGB-D	85.3	-	140
AlexNet, MultiGrasp^29^	RGB-D	88.0	87.1	76
STEM-CaRFs^30^	RGB-D	88.2	87.5	-
GRPN^31^	RGB	88.7	-	200
ResNet-50x2^32^	RGB-D	89.2	88.9	103
GraspNet^33^	RGB-D	90.2	90.6	24
ZF-net^12^	RGB-D	93.2	89.1	-
E2E-net^34^	RGB	98.2	-	63
HTC-Grasp^35^	RGB-D	98.3	96.9	5.4
GR-ConvNet^36^	D	93.2	94.3	19
GR-ConvNet^36^	RGB	96.6	95.5	19
GR-ConvNet^36^	RGB_D	97.7	96.6	20
TF-Grasp^37^	D	95.2	94.9	41.1
	RGB	96.78	95.0	41.3
	RGB-D	97.99	96.7	41.6
**GraspFormer**	RGB-D	**98.91**	**97.30**	**5.1**

**Fig. 4:**
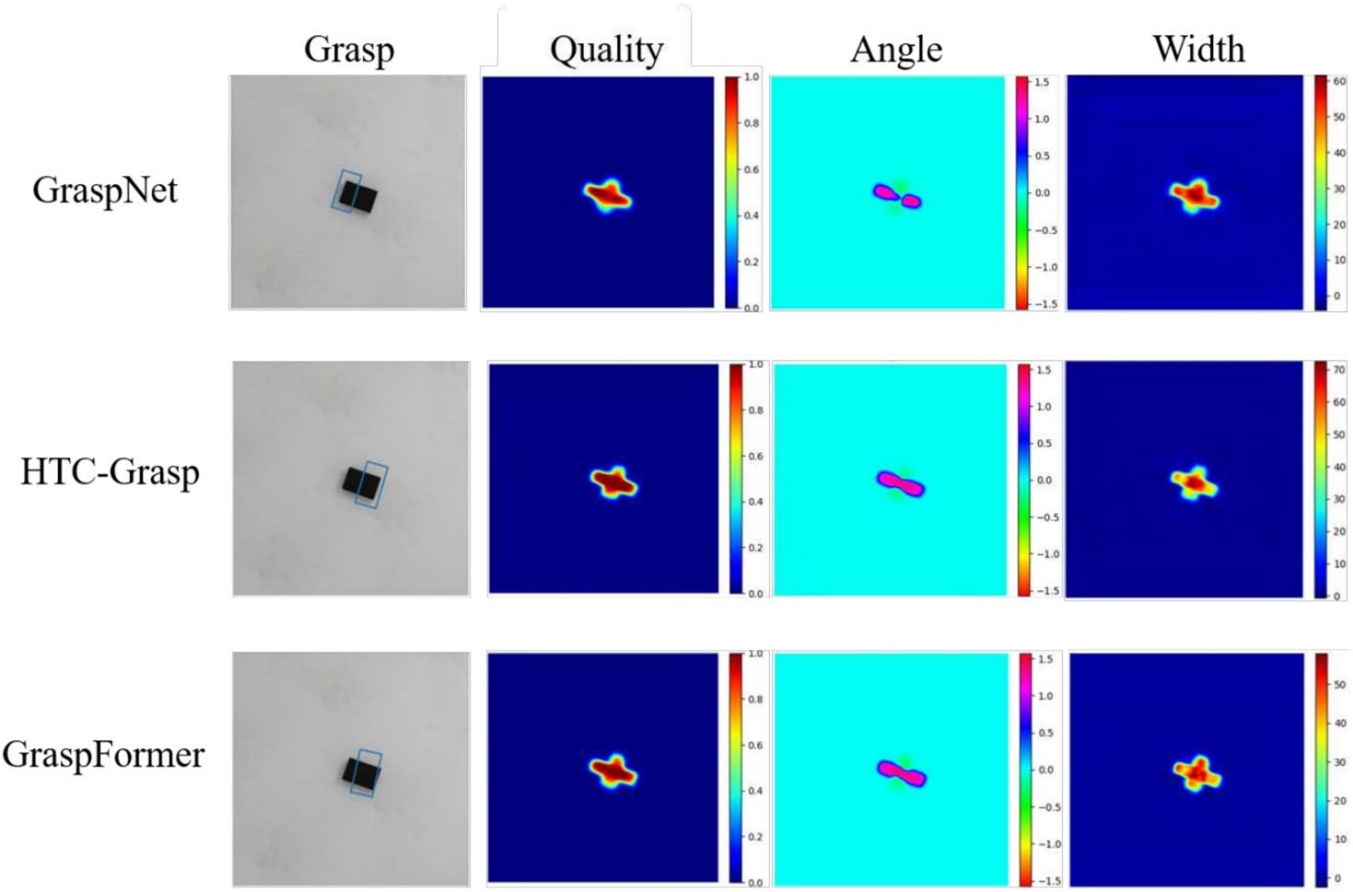
Comparison of the predicted results and heat maps on Jacquard dataset. The columns represent RGB inputs, depth images, grasping rectangles, quality, angle and width heat maps. Note that the bounding boxes are included in the grasp map.

Comparative experiments were also performed using the Jacquard dataset. Figure 4 illustrates examples of predicted heatmaps and grasps from GR-ConvNet, HTC-Grasp, and GraspFormer. The findings reveal that GraspFormer delivers superior grasp quality compared to GR-ConvNet and HTC-Grasp. Table 3 provides the statistical outcomes of GraspFormer on the Jacquard dataset, benchmarked against several classic algorithms. GraspFormer surpassed the other methods, achieving accuracy rates of 96.3%.

**Table 3:** The accuracy on Jacquard grasping dataset.

Method	Input	Accuracy(%)
Jacquard^26^	RGB-D	74.2
GG-CNN2^14^	D	84
FCGN,ResNet-101^13^	RGB	91.8
GQ-STN^38^	D	70.8
ROI-GD^39^	RGB	90.4
Det Seg^34^	RGB	92.59
Det Seg Refine^34^	RGB	92.95
GR-ConvNet^36^	D	93.7
GR-ConvNet^36^	RGB	91.8
GR-ConvNet^36^	RGB-D	94.6
HTC-Grasp^35^	RGB-D	95.8
GraspFormer	RGB-D	**96.3**

Qualitative comparison results for the Cornell and Jacquard datasets are demonstrated in Figure 3 and Figure 4. One can see that:

- As illustrated in the first and third rows of Figure 3 and Figure 4, the GraspNet and HTC-Grasp methods demonstrate low prediction quality in the central area of objects that are easy to grasp. The background predictions by GraspNet and HTC-Grasp are nearly indistinguishable from the actual grasp poses, leading to deviations in the predicted grasp bounding boxes.
- Compared to the Transformer-based HTC-Grasp model, the proposed GraspFormer delivers more accurate predictions of grasp quality and preserves finer shape details. This improvement is achieved by integrating the novel attention mechanism into the Grasp Transformer, which enhances the network’s ability to capture global context and distinguish semantic features. Additionally, the deep channel modulators are incorporated into the framework, enabling the network to learn the importance of each feature channel. This approach optimizes the use of valuable features while minimizing the influence of redundant ones.

**Fig. 5:**
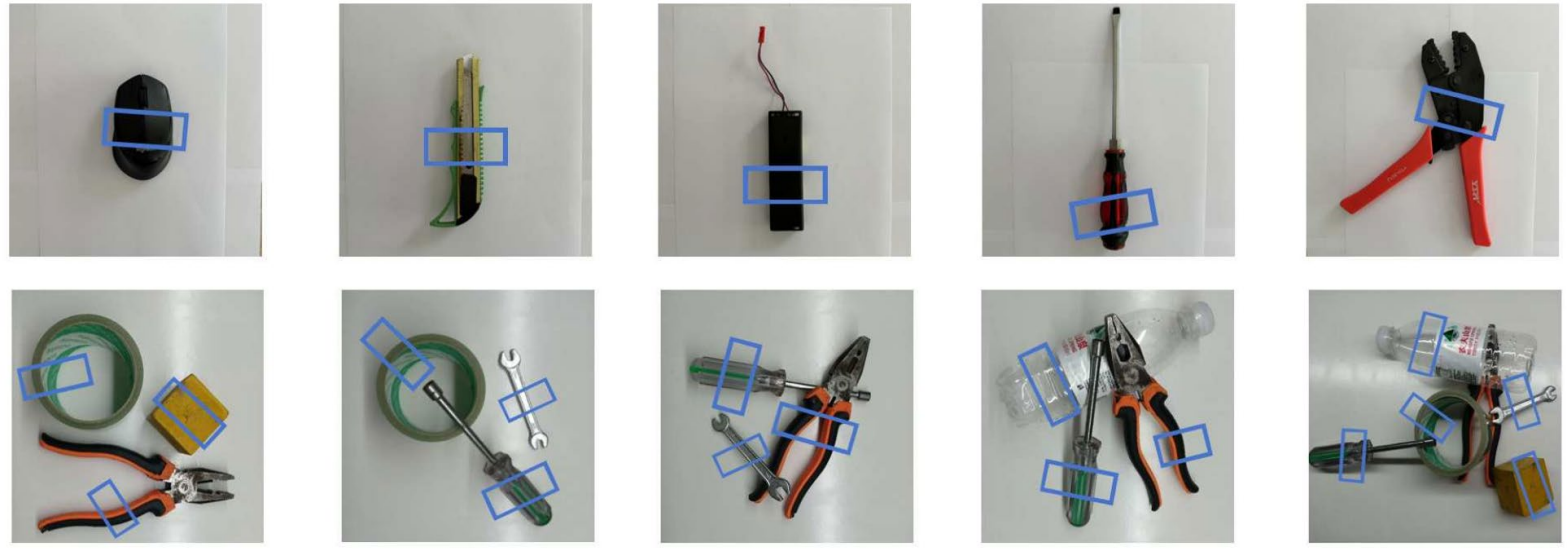
The test result of our method in the real-world multiple objects environment.

The experimental results indicate that our method can accurately pinpoint suitable grasp locations and effectively distinguish graspable areas with a high degree of confidence. As depicted in the third rows of Figure 3, valid grasping pixels are highlighted with scores close to 1, while invalid pixels are assigned lower values. Similarly, in Figure 4, the easily graspable protruding parts of the object are accurately marked with high scores, demonstrating the model’s ability to capture both global context and fine-grained details, such as the precise location and shape of the object. To further assess the efficiency of GraspFormer, experiments were conducted using a test set of images captured by us without additional training. The outcomes shown in Figure 5 demonstrate that the proposed framework can accurately detect grasp regions in an unseen real-world setting.

### Ablation study

In this section, we conduct ablation study on the Cornell dataset to demonstrate the effectiveness of different components in the proposed deep channel modulator.

All ablation studies are conducted on the Cornell dataset to demonstrate the effectiveness of different component in our method. As shown in Table 4, “baseline” is the standard configuration, “$L_{2}\rightarrow L_{1}$” represents that the $L_{2}$-norm is changed to $L_{1}$-norm, “$\frac{\left\| F_{i} \right\| }{\sum _{j=1,2,\dots ,C }\left\| F_{j} \right\| }\rightarrow \frac{1}{\sum _{j=1,2,\dots ,C }\left\| F_{j} \right\| }$” denotes the relative importance is removed, and “$w/skip \rightarrow w/o \hspace{0.5ex} skip$” refers that we delate the skip connection. One can see that only full configuration can achieve best result compared with other variants.

**Table 4:** Quantitative comparisons of ablation study for deep channel modulator.

Case	Accuracy (100%)	
	Image-wise	Object-wise
Baseline	98.91	97.30
$L_{2}\rightarrow L_{1}$	98.74	97.27
$\frac{\left\| F_{i} \right\| }{\sum _{j=1,2,\dots ,C }\left\| F_{j} \right\| }\rightarrow \frac{1}{\sum _{j=1,2,\dots ,C }\left\| F_{j} \right\| }$	98.48	97.11
$w/skip \rightarrow w/o \hspace{0.5ex} skip$	97.85	96.86

### Grasping in realistic scenarios

#### Experimental setup

**Fig. 6:**
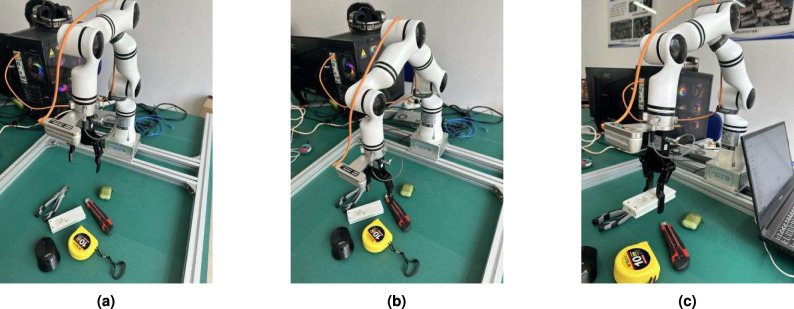
Example of the robotic grasp process. (**a**) shows the initial state of the robot. (**b**) illustrates the robot’s gripper has moved to the target to be grasped. (**c**) shows the state of the object being grasped.

We used an Relman RM65-B robot, a soft parallel manipulator, and an intel realsense d435i camera as part of the experimental setup to design the experiment in this section.The RGB-D camera captures video streams and fed them into the proposed model to obtain the best grasping pose. Subsequently, the robot’s end actuator approaches the target according to the motion planning method, and the gripper is closed to grasp the target.The end actuator is then able to lift the object to another location. Figure 6 illustrates the grasping process.

#### Experiment results

The RGB-D camera captures video streams, which are fed into the proposed model to determine the optimal grasping pose. The robot’s end effector then approaches the target based on the motion planning method, and the gripper closes to grasp the object. Subsequently, the end effector lifts the object to a different location. Figure 6 illustrates the grasping process. In total, 150 grasp attempts were made with household objects, and the robot successfully grasped 141 times, achieving an accuracy rate of 94.0%. Detailed results are presented in Table 5, showcasing the effectiveness of the GraspFormer method in real-world robot grasping tasks.

**Table 5:** Grasp success rates in robotic grasping experiments.

Researchers	Physical Grasp	Success Rate
Lenz^27^	89/100	89.0%
Morrison^14^	110/120	92.0%
Wang^37^	152/165	92.1%
Zhang^35^	168/180	93.3%
**Ours**	141/150	**94.0%**

## Conclusion

In this work, we propose GraspFormer, an encoder-decoder architecture for robotic grasp detection task. In this framework, we design a Grasp Transformer block. It incorporates hierarchical information-guided self-attention to utilize the estimated shallow information and current information to avoid the influence of background features and model the non-local information. Furthermore, we develop an adaptive deep channel modulator to encourage and apply selective in the high-dimensional channel features. These innovations enhance the model’s ability to handle background interference and capture critical features for robust grasp detection. Extensive experiments and ablation studies validate that GraspFormer not only overcomes the shortcomings of previous methods but also achieves performance on par with state-of-the-art solutions. This work demonstrates the potential of Transformer-based approaches in advancing robotic grasp detection and offers a promising direction for future research in this field.

## Data Availability

The datasets generated and/or analysed during the current study are available at https://jacquard.liris.cnrs.fr/ and https://www.kaggle.com/datasets/oneoneliu/cornell-grasp.
